# Constrained DFT for Molecular Junctions

**DOI:** 10.3390/nano12071234

**Published:** 2022-04-06

**Authors:** Linda Angela Zotti, Wynand Dednam, Enrico B. Lombardi, Juan Jose Palacios

**Affiliations:** 1 Departamento de Física Teórica de la Materia Condensada, Universidad Autónoma de Madrid, E-28049 Madrid, Spain; 2 Condensed Matter Physics Center (IFIMAC), Universidad Autónoma de Madrid, E-28049 Madrid, Spain; juanjose.palacios@uam.es; 3 Department of Physics, Science Campus, University of South Africa, Private Bag X6, Florida Park 1710, South Africa; dednaw@unisa.ac.za (W.D.); lombaeb@unisa.ac.za (E.B.L.); 4 Departamento de Física de la Materia Condensada and Instituto Nicolás Cabrera (INC), Universidad Autónoma de Madrid, E-28049 Madrid, Spain

**Keywords:** molecular electronics, constrained density functional theory, benzenediamine

## Abstract

We have explored the use of constrained density functional theory (cDFT) for molecular junctions based on benzenediamine. By elongating the junction, we observe that the energy gap between the ionization potential and the electronic affinity increases with the stretching distance. This is consistent with the trend expected from the electrostatic screening. A more detailed analysis shows how this influences the charge distribution of both the individual metal layers and the molecular atoms. Overall, our work shows that constrained DFT is a powerful tool for studying screening effects in molecular junctions.

## 1. Introduction

Constrained DFT (cDFT) is a powerful tool that has been used for several purposes such as the study of charge-transfer complexes, and tunneling between defects and modeling of reactions [1,2,3,4,5,6,7]. The c-DFT formalism was initially presented by Dederichs et al. [8] and has by now been implemented in various codes, based on either localized basis sets, plane waves or numerical basis functions, mostly making use of Lagrange multipliers [9,10,11,12].

In Ref. [13], some of us presented a version of cDFT that had been implemented in the code ANT [14,15,16,17,18], which is based in turn on Gaussian basis sets. Our implementation was based on different shifts of the Hamiltonian values for different partitions of the system and was successfully used to evaluate the charge neutrality level for a single metal–molecule interface. In the present work, we have extended the use of this implementation to metal–molecule–metal junctions. In particular, we are interested in exploring how the electrostatic screening effects are described upon variation of the metal–molecule distance on each side.

The use of cDFT to study the renormalization of the HOMO-LUMO gap was previously proposed for a planar molecule adsorbed horizontally on a flat surface [19]. There, the SIESTA code was used, which is based on localized atomic orbitals. Charged states were imposed on the molecule by either adding or removing one electron. IP and EA (corresponding to HOMO and LUMO, respectively) were then calculated as differences between total energies of the whole system. The so-evaluated IP and EA were said to be reliable since they were not extracted from the Kohn-Sham eigenvalues but rather obtained from ground-state energies. The results showed that via cDFT it was possible to describe the screening-induced narrowing of the HOMO-LUMO gap obtained upon reduction of the metal–molecule distance. Prompted by this work, we intend to explore if a similar approach can be used for molecular wires.

Understanding the level alignment at metal–molecule interfaces is crucial for achieving control over the transport properties in metal–molecule–metal junctions, energy conversion in organic photovoltaics and many other applications in optoelectronics [20]. The reductions of electron addition and removal energies in molecules when brought close to metal substrates has been observed in various experiments [21,22,23,24]. In fact, the original gap between the molecular ionization potential (IP) and electron affinity (EA) is known to be renormalized due to image-charge effects, charge transfers and hybridization. In particular, the first effect consists of the Coulomb metal–molecule interaction arising from adding or removing an electron in the molecule and from the ensuing polarization, which causes the IP-EA gap to narrow. This effect is not included in standard DFT, mainly due to the incorrect interpretation of Kohn-Sham eigenvalues as quasiparticle excitation energies.

A proper description of the electronic properties can be given by the many-body perturbation theory within the GW approximation [25,26], (although it was shown [27] that conventional GW needs refinement to reproduce the correct alignment). However, this method is known to be very time consuming, and the need for different strategies has become unquestionable. In the field of charged defects, for instance, the quest for a proper treatment of screening effects is very active, and various techniques have been proposed, especially concerning the use of periodic boundary conditions [28]. In the DFT + Σ, one of the most commonly used techniques developed over the years to correct the HOMO-LUMO gap in molecular junctions, screening effects are introduced via a classic electrostatic model [29,30]. Although very useful, this technique involves defining the position of image-charge planes and a reference system, which in certain cases may not be so straightforward [31,32]. Moreover, the model involves assuming perfectly conducting planes, which, in certain cases, is far from the shape of realistic electrodes. Last but not least, the simple model neglects the internal screening response of the molecule to the polarization of the metal surface, which is small for a flat molecule oriented parallel to the surface but not negligible for a polarizability perpendicular to the surface [33]. Therefore, it would be interesting to explore how screening effects are described by cDFT, which should not be affected by these problems. For the sake of completeness, it is important to mention that other approaches have also been proposed to take screening effects into account. For instance, in Ref. [34], the IP-EA gap reduction was evaluated by introducing the polarization-induced potential in the self-consistent cycle of DFT. This potential was obtained via a solution to the Poisson’s equation, where the polarizable environments were replaced by their classical electrostatic energies. However, it was stated that such an approximation neglects the kinetic energy and the exchange-correlation energy associated with the charge built up in the polarized metal. Thus, it would be interesting to explore other techniques such as cDFT, which we proceed to discuss in the following sections.

## 2. Methods

Our method was implemented in the code ANT, Ref. [15] which is built as an interface to Gaussian [35] and employs Green’s function techniques as well as parametrized tight-binding Bethe lattices in the electrode description. By connecting a Bethe lattice to the metal cluster, a new system is built, where the molecule is now connected to a semi-infinite electrode (see Ref. [15] for more details). In the spirit of the Landauer formalism, a metal–molecule–metal junction is divided into three parts, namely, the semi-infinite left (L) and right (R) leads and the central area, from here on called the device. The density matrix of this region is calculated as

(1) $$P\left(\mu \right)=-\frac{2}{\pi }Im\int_{-\infty }^{0}dEG^{+}\left(E;\mu \right)$$

where the retarded Green’s function is given by

(2) $$G^{+}\left(E;\mu \right)={\left[\left(E-\mu \right)\cdot S-H-\Sigma \left(E,\mu \right)\right]}^{-1}.$$

Here, *H* is the Hamiltonian of the main region; Σ is the lead self energy, which describes the coupling to the semi-infinite lead; and μ is a quantity by which the on-site energies of the Hamiltonian must be shifted in order to ensure the total-charge neutrality (μ is opposite in sign to the chemical potential).

The cDFT method used for the present work was implemented as an extension of the procedure we used successfully in a previous work for a single metal–molecule interface [13] to evaluate the charge neutrality level. For the study of a metal–molecule–metal junction, we now divide the system into three parts. Any charge transfer between the three parts is forbidden. Both leads and molecules were forced to keep a specific number of electrons. To this aim, μ was computed in alternation so as to meet the imposed charge constraints on the two metal electrodes and the molecule. If $N_{L}$($N_{R}$) and $N_{mol}$ are the desired number of electrons in the left (right) electrode and in the molecule, respectively, then three different local potentials $\mu_{L}$, $\mu_{mol}$ and $\mu_{R}$ are calculated so that

(3) $$Tr\left[\left(P_{L}\left(\mu_{L}\right)S\right)\right]=\sum_{i,j=1}^{NAO_{L}}P\left(i,j\right)S\left(i,j\right)=N_{L}$$

(4) $$Tr\left[\left(P_{mol}\left(\mu_{mol}\right)S\right)\right]=\sum_{i,j=NAO_{L}+1}^{NAO_{L}+NAO_{mol}}P\left(i,j\right)S\left(i,j\right)=N_{mol},$$

(5) $$Tr\left[\left(P_{R}\left(\mu_{R}\right)S\right)\right]=\sum_{i,j=1}^{NAO_{R}}P\left(i,j\right)S\left(i,j\right)=N_{R}$$

where $NAO_{L(R)}$ and $NAO_{mol}$ are the number of atomic orbitals of the left (right) electrode and the molecule, respectively.

Note that these charges were calculated in the spirit of the Mulliken partition. For the sake of comparison, we also implemented a Löwdin partition, where the individual charges for a generic number of atomic orbitals NAO are obtained as $\sum_{i,j=1^{NAO}}S{\left(i,j\right)}^{1/2}P\left(i,j\right)S{\left(i,j\right)}^{1/2}$

At each iteration step the density matrix $P^{o}$ is then built as a block matrix as follows:

(6) $$P^{o}=\left(\begin{array}{ccc} P_{L}\left(\mu_{L}\right) & P_{L-mol} & P_{L-R}\\ P_{L-mol} & P_{mol}\left(\mu_{mol}\right) & P_{R-mol}\\ P_{L-R} & P_{R-mol} & P_{R}\left(\mu_{R}\right) \end{array}\right)$$

The off-diagonal terms in the submatrices $P_{R-mol}$, $P_{L-mol}$ and $P_{L-R}$ were set to zero. The so-built density matrix is out of equilibrium, with three different local potentials in the metal leads and the molecule, consequently giving rise to a step potential across the system.

**Figure 1:**
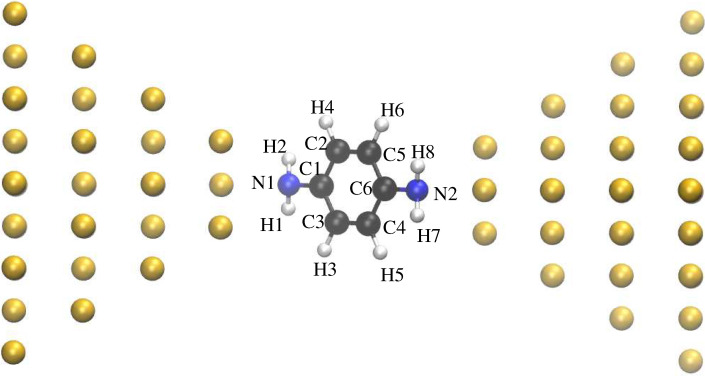
Junction incorporating BDA between two Au34 clusters.

For the calculations presented in this work, we used the PBE exchange-correlation functional [36], and a LANL2DZ basis set [37] for all atoms but those in the outermost layer on each side, for which we used a CRENBS basis set [38]. To test our implementation of cDFT, we chose benzenediamine (BDA) as the molecule held between the two electrodes in the junction because several theoretical studies on its level alignment are available in the literature for comparison, as well as experimental data from photoemission experiments [32,39,40,41,42]. In Figure 1 we show the structure we analyzed, which incorporates BDA bound to the two Au34 clusters on each side, in a binding geometry similar to that analyzed in Ref. [41]. The ionization potential and electron affinity were calculated as IP = E(N − 1) − E(N) and EA = E(N) − E(N + 1), respectively, where N is the gas-phase total number of electrons in the molecule. E(N − 1), E(N) and E(N + 1) are the total energies obtained by cDFT calculations in which $N_{mol}$ was set to N − 1, N and N + 1, respectively. For the sake of simplicity, we will identify the LUMO as -EA and the HOMO as -IP.

## 3. Results and Discussion

**Figure 2:**
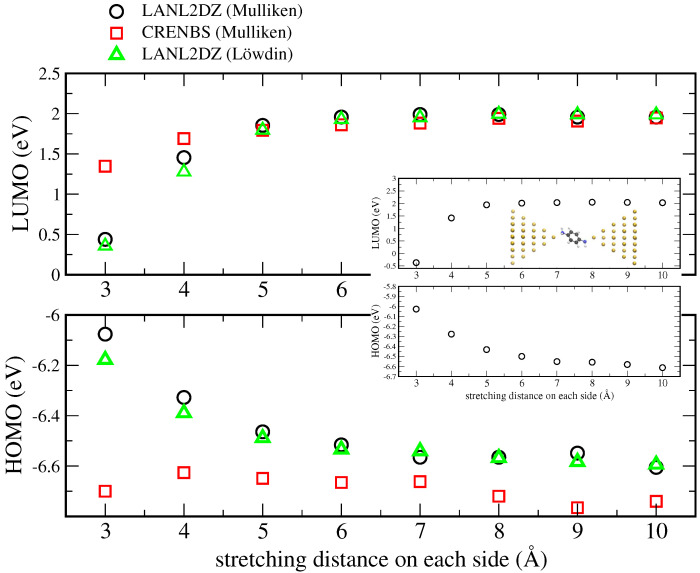
LUMO (**upper panel**) and HOMO (**lower panel**) obtained for the geometry shown in Figure 1 using the Mulliken partition with the mixed LANL2DZ-CRENBS basis sets (black circles) and CRENBS on all atoms (red squares), and for the Löwdin partition on the mixed LANL2DZ-CRENBS basis sets (green triangles). The inset shows the curves obtained using the Mulliken partition and the LANL2DZ basis set for the top-binding geometry there depicted.

In Figure 2, we report the values obtained for HOMO and LUMO upon increasing the metal–molecule distance on each side in a stepwise manner. As our setting the off-diagonal terms to zero is expected to increase inaccuracies at shorter distance, we only considered the range 3–10 Å. For this, we considered three different cases: cDFT calculations in which the Mulliken partition was applied, with a mixed LANL2DZ-CRENBS basis sets (which will be henceforth called LANL2DZ for brevity) and with CRENBS basis sets for all atoms, and Löwdin partiton with the mixed LANL2DZ-CRENBS basis sets.

We observe that the use of LANL2DZ results into the expected narrowing of the gap for shorter distances for both types of partitions. The same does not apply to the CRENBS values. This is probably due to the fact that the latter contains a very low number of basis set functions, thus highlighting the fact that a more complete basis set (such as LANL2DZ) is needed in order to describe screening effects. In the case of the HOMO, its position is not shifted to higher values for shorter distances as expected but rather oscillates around a constant value. Interestingly, the LUMO shows a smaller variation than that obtained with LANL2DZ. In the framework of a classical image-charge model, this would correspond to a position of the image charge plane farther from the molecule [32].

Note that the equilibrium electronic configuration would involve a charge transfer from the molecule onto the electrodes, which would increase with the distance. This would result in the formation of an additional dipole at the interface, which may affect the overall level renormalization. Consequently, the total level shift induced by screening should not be compared directly with that obtained by other techniques (such as DFT + Σ, for instance), where the corresponding contribution is obtained for junctions in which the molecule has already donated/accepted charge to/from the metal [29,30,41]. For the sake of clarity and with the aim of discerning the effect of imposing different charge states on the molecule solely, this has not been taken into account in the present work.

In Figure 2, we also observe that, for a stretching distance of 10 Å, the LUMO and HOMO values obtained with the LANL2DZ basis set are quite close to those obtained in the gas phase (−6.7 eV and 1.95 eV, respectively). The latter were obtained by standard DFT calculations on the isolated molecule, more specifically by subtracting the ground state energies obtained for the ±1 and 0 charge states. The inset in Figure 2 shows the energy shift of HOMO and LUMO for a different binding geometry (obtained with the Mulliken partitioning and the LANL2DZ basis set), in which the molecule is comprised between two Au35 clusters, both terminating with a single apex atom. Interestingly, we also notice that, in our calculations, imposing the charge neutrality of the whole system (as was necessary to do in Ref. [19]) actually led to inaccurate results. We ascribe this difference to the lack of cell periodicity in our model.

**Figure 3:**
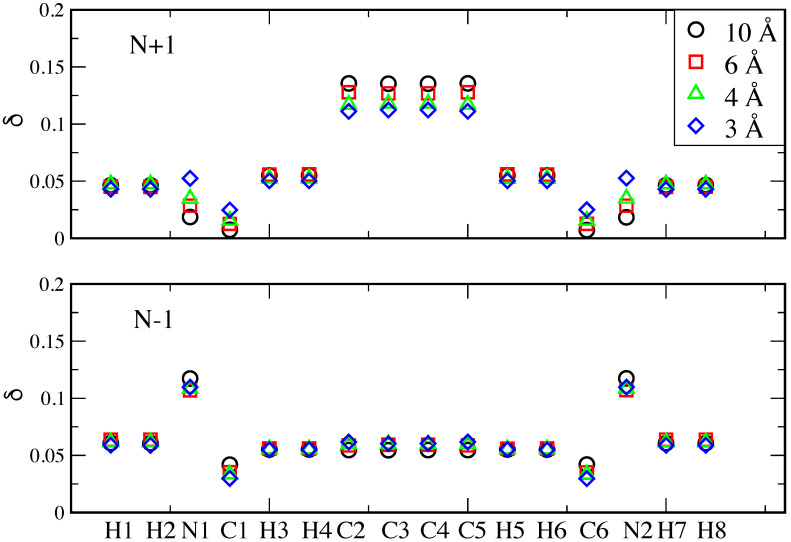
Distribution of the increased negative (**upper panel**) and increased positive (**lower panel**) charge on each atom. On the horizontal axis, the position of the atoms’ labels follows that of the corresponding atoms along the junction of Figure 1 from left to right.

**Figure 4:**
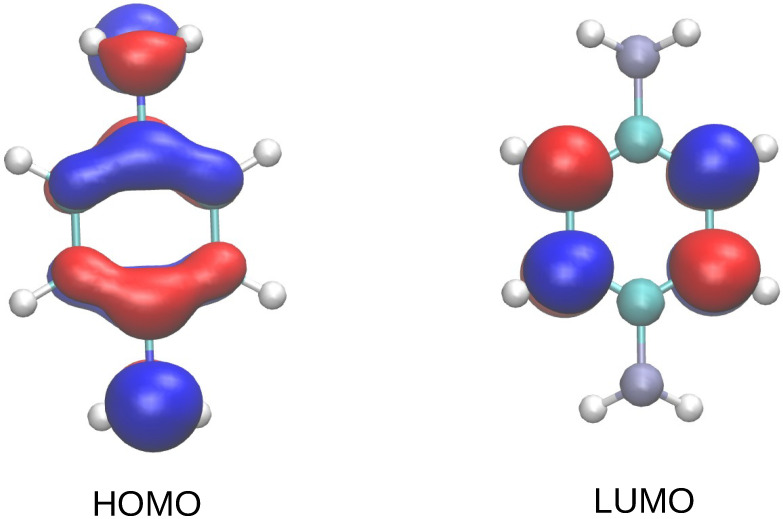
Spatial distribution of HOMO and LUMO for the BDA molecule.

We now turn to gaining deeper insight into the charge distribution within the molecule. In Figure 3, we show the variation of electron charge on the individual atoms at four selected stretching distances for the N + 1 and N − 1 cases with respect to the N case. We observe that such a variation reflects the different localization of the gas-phase frontier orbitals (depicted in Figure 4). While the HOMO is located on both the phenyl ring and the anchoring groups, the LUMO is mainly located on four C atoms of the phenyl ring. Consequently, the electron addition in the N + 1 case mostly involves these four C atoms, while a significant variation on the N atoms only takes place in the N − 1 case. As mentioned above, the approximations our method are based on are expected to produce inaccuracies at short metal–molecule distances. A more refined procedure will be tackled in future works to address the off-diagonal terms, as well as different kinds of system partitioning [43]. Examples of possible different charge partitions to explore include Hirshfeld, CM5, MK, ChElPG, MBS, NPA, DDEC, LoProp and Bader charges [44].

**Figure 5:**
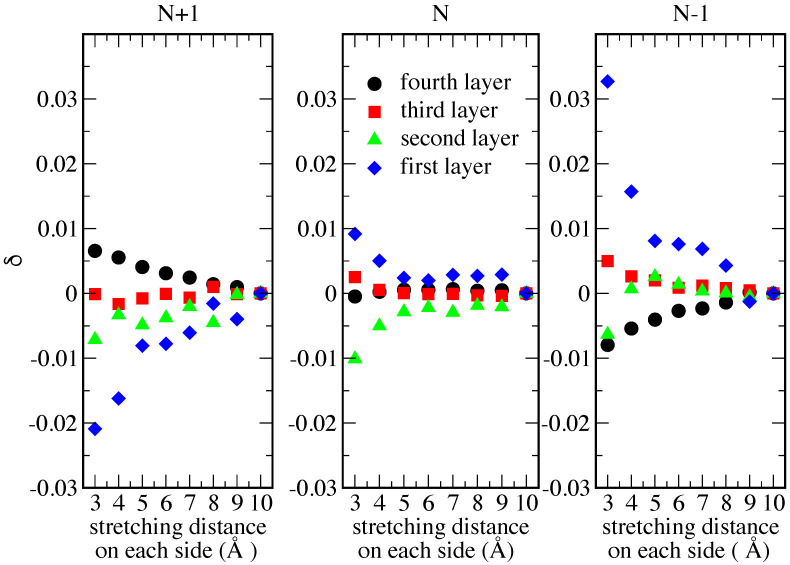
Variation of the number of electrons (evaluated with respect to the 10 Å value) in each of the metal layers obtained for each stretching distance and for each charge state (N + 1, N and N − 1, N being the gas-phase number of electrons in the molecule). Layers are numbered from the one closer to the molecule (first) to the one farther from it (fourth).

Finally, in Figure 5, we show the variation δ of the number of electrons (with respect to the 10 Å value) in each of the metal layers, obtained for each stretching distance and for each charge state (N + 1, N and N − 1, N being the number of electrons in the molecule). For each layer, the corresponding δ value was obtained by averaging the variations on all atoms of that specific layer. As expected, we observe that, as the distance decreases, the absolute value of δ increases. In the N + 1 case, the number of electrons in the first layer (closer to the molecule, blue diamonds) decreases as a screening response to the increased negative charge on the molecule. Consequently, the number of electrons in the last layer (farther from the molecule, black dots) increases. The opposite occurs for the N − 1 case. As for the N case, no charge variation is detected in the layer farther from the molecule; conversely, in the two layers closer to the molecule, the same trend as for the N + 1 case is observed, although with a lower δ value at short stretching distance in the first layer.

## 4. Conclusions

We found that cDFT is a powerful tool for analyzing the molecular level renormalization induced by electrostatic screening in molecular junctions. Our model is based on approximating off-diagonal terms of the density matrix to zero, and thus it is expected to lead to certain inaccuracies at shorter metal–molecule distance. This issue will be tackled in future works. Nevertheless, our results clearly show that a more refined implementation of the cDFT capable of dealing with this limitation should be able to provide a quantitative evaluation of the screening-induced level realignment. Moreover we envisage that, by including also the formation of dipoles arising due to charge transfer at the metal–molecule interface, this approach should ultimately make it possible to quantify the energy realignment of the frontier orbitals in molecular junctions at the contact regime. This could be very useful as it would take into account the actual structure of the clusters (rather than making use of perfectly conducting planes, for instance). It would also make it simple to extract the screening contribution for any type of positioning of the molecule with respect to the clusters. The development of a model that takes into account all these factors will be the object of future works.

## Data Availability

All the data and additional information supporting the findings of this study are available from the corresponding authors upon reasonable request.

## Abbreviations

The following abbreviations are used in this manuscript:

BDA	benzene diamine
cDFT	constrained density functional theory
HOMO	highest occupied molecular orbital
LUMO	lowest occupied molecular orbital
